# Perceptions of Polish and German physiotherapists about their professional education and development: a cross-sectional nonrandomized questionnaire cohort study

**DOI:** 10.1186/s12909-022-03619-w

**Published:** 2022-07-15

**Authors:** Krzysztof Handkiewicz, Mariusz Drużbicki, Agnieszka Guzik, Artur Stachura, Małgorzata Makiewicz

**Affiliations:** 1 Health and Sports Center ‘Aquadrom’, Graal-Müritz, Germany; 2 Department of Physiotherapy, Institute of Health Sciences, Medical College, University of Rzeszow, Rzeszów, Poland; 3 Koszalin University of Technology, Koszalin, Poland; 4 Maria Grzegorzewska Academy of Special Education Warsaw, Warsaw, Poland

**Keywords:** Physiotherapist, Professional education, Professional development, Professional career, Poland, Germany

## Abstract

**Background:**

The approach to the education and professional advancement of physiotherapists is particularly relevant today. To date, no studies have compared the perceptions of physiotherapists regarding professional issues in geographically close European countries such as Poland and Germany. Therefore, this study’s purpose was to compare Polish and German physiotherapists’ perceptions related to their profession, entry-level education, and career opportunities.

**Methods:**

We recruited 565 physiotherapists from Poland and 560 physiotherapists from Germany. An opinion polling method based on a questionnaire was applied in the study. The survey was conducted in 48 facilities located throughout the territories of the two countries. The assessment focused on the following three issues: 1) professional education (form and content of educational programs, organizational aspects, and effects of education); 2) professional development and career opportunities; and 3) the relationship between years of service and perceptions of professional education, career satisfaction and advancement opportunities.

**Results:**

German respondents rated specific aspects of their education, development opportunities and professional careers more highly than their Polish counterparts (*p* = 0.001). A highly significant negative correlation was identified in both groups between all the assessed aspects of professional education and years of service (0.9 ≤ |R| < 1, *p* = 0.001).

**Conclusions:**

Opinions on their professions expressed by physiotherapists from closely neighboring countries, namely, Poland and Germany, were surprisingly disparate. Compared to their Polish colleagues, German physiotherapists viewed their experiences more favorably vis-a-vis entry-level education, career opportunities, and professional status. Further study is needed to establish whether these findings reflect actual differences, sampling bias, or other factors.

## Background

Physiotherapy is recognized as a health care profession in all European Union (EU) countries [1]. Consequently, researchers have been investigating various aspects of formal physiotherapy education and the professional advancement of physiotherapists. In addition to theoretical and practical components of teaching curricula, clinical education is an integral part of the courses preparing physiotherapy students for the job. Clinical training plays an important role in building professional expertise and in combining practice with adequate substance-related foundations [2, 3].

Studies evaluating physiotherapy courses and teaching curricula aim to identify the most effective educational methods and existing deficiencies [4]. For example, based on student, educator and expert opinions, researchers reported there were needs for closer cooperation between health care workers and patients [5]; ongoing, evidence-based updates regarding physiotherapy interventions [6–9]; and cooperation between practicing physiotherapists and researchers carrying out empirical studies [10, 11]. The related findings also show that it is necessary to consider international experiences [12] and the cultural specificity of learners [13] in designing local teaching curricula and to take into account the preferred learning methods of students in combination with their on-the-job training [14]. Furthermore, educational programs for physiotherapists must provide various ways to solve patient-related problems and develop students’ interpersonal and social skills [15, 16] and complex competencies [17].

Poland and Germany, two neighboring countries located in central Europe, in the course of history have not always had a good relationship, but over the years, the countries have become closer to each other in many respects. This is reflected by the partnership within the EU and by a variety of joint projects carried out in scientific, social, economic and other domains [18]. The physiotherapist profession is recognized in both countries as an independent medical profession subject to legal regulations. Consequently, the profession gained a higher status in both countries. The use of the title “physiotherapist” is protected by the law [19]. In Germany, the first federal regulations applicable to those performing the profession of physiotherapy were introduced in 1959, when German professional organizations joined World Physiotherapy (formerly the World Confederation for Physical Therapy). The first detailed rules for professional physiotherapy practice in Germany were specified 20 years later. These laws are in force today, as amended by the specific Bundesland authorities [20]. In Poland, the first nationwide organization was started in 1967 under the name the Polish Society of Physiotherapists, and it has cooperated with World Physiotherapy since that time. However, it was not until 2015 that laws for the profession of physiotherapy were adopted by the Polish Parliament. Currently, the largest professional organization in the country is the Polish Chamber of Physiotherapists, which joined World Physiotherapy in 2020 [21]. Differences and similarities between the physiotherapy professions in Poland and Germany are presented in Table 1.

**Table 1:** Differences and similarities in the profession of physiotherapy in Poland and Germany

Characteristics of physiotherapy profession	Poland	Germany
**Number of schools/programs**	66 (40 public, 26 non-public entities)	A total of 122 vocational programs and undergraduate courses (14 undergraduate courses in non-public entities, with State exam 108 vocational schools, private and state-owned)
**Accreditation**	Required	Required
**Accreditation body**	Polska Komisja Akredytacyjna (Polish Accreditation Committee)	Kultusministerkonferenz (Standing Conference of the Ministers of Education and Cultural Affairs) Each of the 16 federal states (Bundesland) has its own specific requirements. Educational programs do not always comply with curricula adopted in other federal states.
**Professional bodies**	Polskie Towarzystwo Fizjoterapii (Polish Society of Physiotherapists), Stowarzyszenie Fizjoterapia Polska (Polish Physiotherapy Association), Polskie Stowarzyszenie Specjalistów Fizjoterapii (Polish Association of Physiotherapy Specialists), Krajowa Izba Fizjoterapii (Polish Chamber of Physiotherapists)	Physio Deutschland (Physio Germany) – nationwide organization with separate branches in 16 German states, Vpt – Verband fur Physiotherapie (Association for Physiotherapy) VDB – Physiotherapieverband (Physiotherapy Association) – these are private entities
**Licensing bodies**	Polish Chamber of Physiotherapists	Ministry for Social Affairs and Health, specific to each federal state; after documents are received from the school confirming positive result in theoretical and practical state examination.
**Requirements for licensing**	From 2019, status of physiotherapist is awarded to individuals who completed 5-year Master degree courses, or 3-year undergraduate and 2-year graduate courses.	Status of physiotherapist is awarded to those who have completed 3-year vocational course at Berufschule or undergraduate course, who has passed practical and theoretical state exam.
**Requirement for continuing professional education**	No specific requirements; self-selected specialisation in physiotherapy (senior assistant in physiotherapy) completed as a post-graduate course	No specific requirements; self-selected specialisation in physiotherapy completed in courses conducted by vocational schools and undergraduate programs.

However, knowledge of the differences and similarities between the education systems and professional development of physiotherapists in Poland and Germany is insufficient. No studies have yet been conducted in this area. The related research findings may provide important information and enable professional organizations to standardize the educational systems and organization of physiotherapy practice in neighboring EU countries.

This study aimed to assess similarities and differences in the systems of education and professional development of physiotherapists in Poland and Germany based on their perceptions. The following three research questions were formulated:

- Q1) Based on the opinions of Polish and German physiotherapists, how do the systems of entry-level physiotherapy education in the two countries compare?
- Q2) According to participants’ perceptions, are there differences between the two countries in the professional status of physiotherapy as well as related career opportunities and working conditions?
- Q3) Do years of service impact Polish and German physiotherapists’ perceptions of their formal education, job satisfaction and career opportunities?

## Methods

### Participants and setting

The group enrolled for the principal part of the study included 565 physiotherapists from Poland and 560 physiotherapists from Germany. The Polish group comprised 192 (33.98%) women and 373 (66.02%) men, and the German group consisted of 79 (14.11%) women and 481 (85.89%) men. Polish and German participants of the study on average were aged 36.48 (SD 11.22) years and 42.83 (SD 17.98), respectively. The participants’ characteristics are presented in Table 2.

**Table 2:** Years of service and gender of physiotherapists participating in the study

Years of service	Polish therapists				German therapists			
	Women		Men		Women		Men	
	Number	%	Number	%	Number	%	Number	%
below 1	23	12.0	160	42.9	0	0.00	0	0.00
1	0	0.00	106	28.4	0	0.00	0	0.00
2–4	0	0.00	77	20.6	0	0.00	0	0.00
5–6	0	0.00	4	1.1	0	0.00	72	15.0
7–10	6	3.1	26	7.0	0	0.00	88	18.3
11–15	12	6.3	0	0.00	0	0.00	131	27.2
16–20	3	1.6	0	0.00	4	5.1	190	39.5
over 20	148	77.1	0	0.00	75	94.9	0	0.00
**Total**	**192**	**100.00**	**373**	**100.00**	**79**	**100.00**	**481**	**100.00**

The study was designed to enable conclusions related to the entire statistical population based on the information collected from a representative sample. A minimum size of a sample representative for the community of physiotherapists was calculated for this purpose. The sample size was defined at a confidence level of a = 0.95, with a fraction size of 0.7 and a maximum error of 0.04. The minimum sample size for Polish physiotherapists was defined as 500 participants and for German physiotherapists as 502 participants. The study involved 565 participants from Poland and 560 individuals from Germany.

### Study protocol

The cross-sectional nonrandomized cohort study was carried out in two stages in 2017–2018. A pilot study was conducted at the first stage and was followed by the principal part of the study. The project received approval from the University of Rzeszów’s Bioethical Commission. Experimental conditions conformed to the policies and mandates of the Declaration of Helsinki.

### Procedure and measures

#### Pilot study

In view of the fact that the study was designed to apply a questionnaire developed by the authors, the accuracy of the tool was verified in a pilot study. The set of questions was prepared in the German language in cooperation with German physiotherapy specialists and an orthopedic surgeon. Consultations with these experts made it possible to refine the content of the questionnaire. The translation and cultural adaptation process consisted of a few stages [22]. The first stage involved a forward translation. Two translations were made from the original (German) to the target (Polish) language. As a result, it was possible to compare the translations for any discrepancies; poorer wording choices were identified and resolved in a discussion between the two translators. The second stage involved a synthesis of the translations, i.e., based on the original questionnaire and the two versions provided by the translators, a combined version of the questionnaire was produced. In the third stage, a back translation of the questionnaire was made (to the original language) to ensure that the translated version conveys the same meanings as the original version. Subsequently, the expert committee (methodologist, Polish and German physiotherapists and translators) consolidated all the versions of the questionnaire into the final version of the tool. The questionnaire prepared this way was then used in the pilot study, during which responses were collected from 60 physiotherapists (30 each from Germany and Poland). By conducting the preliminary study with 60 respondents, it was possible to validate the cultural and language adaptation of the specifically designed questionnaire and to confirm that it was accurately understood by the study participants and would enable effective assessment in a larger study group. In the process of preparing the manuscript, the Polish version of the questionnaire was translated into English by a professional translator.

### Outcome measures

The study applied an opinion polling method and was based on a survey that was carried out using a specifically designed questionnaire. The research tool consisted of 50 questions divided into the following three thematic groups:

1. Sociodemographic profile comprising 11 questions (related to topics such as age, gender, years of service, residence and place of employment).
2. Evaluation of physiotherapy education comprising 23 questions (related to issues such as conditions at university, curriculum, functioning of the university, competencies acquired during the university course).
3. Evaluation of professional development and career opportunities comprising 16 questions (related to issues such as the participant’s continuing professional development, conditions at work and the future of physiotherapy as a profession).

### Procedures

At the time the study was initiated and carried out, random selection of the Polish participants was impossible because the databases of the Polish Chamber of Physiotherapists were still under construction. Additionally, in Germany, there is no organization associating physiotherapists in the country; hence, it was also impossible to randomly select a group of participants. Therefore, to recruit participants for the study, online databases were reviewed to identify entities employing physiotherapists, and 52 facilities were selected each in Poland and Germany. Subsequently, letters were sent to these entities via Poczta Polska and Deutsche Post asking whether they agreed to participate in an anonymous survey. No responses were sent back by 42 entities, and 14 facilities refused to participate in the survey, whereas 48 entities agreed to take part. A request for consent to participate in the study was sent to the facilities that agreed. This recruitment procedure covered the whole territory of the two countries. The survey forms were sent via Poczta Polska and Deutsche Post.

Because of the insufficient number of participants recruited, a request was sent via e-mail to the Polish and German administrators of social networking sites for professional groups including physiotherapists. The purpose was to recruit an appropriate number of participants, in line with the calculated statistically adequate sample size. Health care entities (hospitals, spa facilities, clinics) providing physiotherapy-related services were selected in both countries. In addition to physiotherapists working for public entities, the study participants included self-employed private practitioners. In addition to these traditional methods of recruitment, the survey was also conducted among physiotherapists active on online social networking sites. For this purpose, approval was obtained from google.pl and google.de administrators.

### Data analysis

Data analysis was performed using the program SPSS Statistics. Distributions of the investigated variables were examined for normality using the Shapiro–Wilk W-test. Regarding the physiotherapists’ perceptions of their formal education, professional development and career opportunities, comparative analysis took into account median values of the ratings in the specific questions by the two groups of respondents; the significance of the differences in the ratings between Polish and German respondents was assessed using the Mann–Whitney U test. The relationship between the respondents’ years of service and their perceptions of their formal education, career opportunities, and job satisfaction was assessed using Spearman’s rank correlation coefficient. At the last stage, regression analysis was carried out taking into account the variables describing the participants’ perceptions of the educational process and professional career in relation to years of service and, additionally, age of the respondents. For the needs of this analysis, it was assumed that the rating scale used in the research tool was a linear scale. Statistical significance was assumed for values of *p* < 0.05.

## Results

### Q1. Rating of entry-level physiotherapy education systems by the groups of respondents

Based on their own experience, the respondents assessed three aspects of formal physiotherapy education systems: 1) the teaching/learning component, i.e., the form and content of curricula, 2) organizational issues, and 3) effectiveness of education. German respondents commonly expressed more favorable perceptions of specific aspects of their education than their Polish counterparts. Only three aspects of physiotherapy education received higher ratings from Polish respondents: opportunities to study abroad, practical education for the profession and the system of internships. In these items, the analysis showed higher median values of the ratings awarded by Polish participants than those of German respondents. On the other items, the German education system received higher ratings. Ultimately, according to participants’ perceptions, the two entry-level physiotherapy education systems differed in all the evaluated characteristics, with most aspects of the Polish system receiving lower ratings. The significance of the differences between the ratings awarded by Polish and German respondents was confirmed by the result of the Mann–Whitney test (*p* = 0.001). Notably, the responses provided by German participants did not include low ratings (below 4 on a six-point scale), whereas some aspects of the Polish education system received the lowest possible rating from many participants. This tendency is most visible in the perceptions related to student facilities, which German respondents rated as “good” (13.9%) or “very good” (86.1%); these highest ratings were used by only 20.4% of the Polish respondents, and 6% of Poles reported that student facilities were “poor” or “very poor”. This distribution of responses may result from cultural differences between the two nations (Table 3).

**Table 3:** Rating for physiotherapy education systems by German and Polish respondents (rating on a scale from the lowest 1 to the highest 6)

Characteristics of physiotherapy education systems	Polish physiotherapists			German physiotherapists		
	Min.	Median	Max.	Min.	Median	Max.
**I. Form and content of educational programs**						
Quality of teaching	2	5	6	5	6	6
Specialties available in physiotherapy courses	2	5	6	5	6	6
Work by the academic stuff	2	5	6	5	6	6
Teaching skills of the academic staff	2	5	6	3	6	6
Curriculum	2	4	6	2	6	6
System of internships	1	5	6	4	5	6
**II. Organisation of education**						
Conditions at the university	2	5	6	4	6	6
Opportunity to do part of the course abroad	2	4	6	3	3	4
Functioning of university library and reading room	2	4	6	4	5	5
University administration and functioning	2	5	6	4	6	6
Student facilities at the university	1	4	6	5	6	6
**III. Effectiveness of education**						
Acquisition of knowledge-related professional qualifications	2	5	6	4	6	6
Acquisition of skills-related professional qualifications	2	5	6	5	6	6
Acquisition of professional qualifications in the area of social competences	2	5	6	4	6	6
Practical training for the profession	1	6	6	5	5	6
Acquisition of qualifications relative to current needs of labour market	1	6	6	5	6	6

### Q2. Professional development and career

Participants provided responses to five questions related to aspects of their job (atmosphere at work, relations with their supervisor, salary, job satisfaction and professional career), which they rated on a 10-point scale. German respondents provided a higher rating in each of the five aspects, reflecting more favorable perceptions of their job than Polish respondents. As in the case of the first research question, median values of the ratings in the specific items were compared, and significance of the differences between the perceptions of Polish and German respondents was examined using the Mann–Whitney U test (at a significance level of *p* = 0.001). The largest difference can be observed in responses to the question about career advancement; the median value of the ratings by Polish respondents was 6, less than the median of 9 in the case of German respondents (on a 10-point scale). For their evaluation of their formal education, German respondents used high ratings (7–9) far more often than Polish respondents, and they did not use ratings below 5 at all. Conversely, many Polish respondents selected very low or low ratings, e.g., in question 48, ratings between 1 and 4 accounted for 33.8% of the responses in the Polish group, while no German respondent selected a rating lower than 5 (Table 4).

**Table 4:** Evaluation of their situation at work, by Polish and German physiotherapists (rating on a scale from the lowest 1 to the highest 10)

Work-related issues	Polish physiotherapists			German physiotherapists		
	Min.	Median	Max.	Min.	Median	Max.
Atmosphere at work	1	9	10	5	10	10
Satisfying relations with supervisor	1	7	10	6	9	10
Salary satisfaction	1	7	10	8	9	10
Job satisfaction	1	8	10	9	10	10
Career advancement	1	6	10	5	9	10

### Q3. Relationship between years of service and respondents’ perceptions of their formal professional education, job satisfaction and career opportunities

The findings show a strong statistically significant negative correlation between participants’ perceptions of all the aspects of physiotherapy education and reported years of service (all correlations are significant at a level of *p* = 0.001). This shows that therapists with more work experience tend to present less favorable perceptions of their formal education than their more inexperienced counterparts. This is true for both Polish and German respondents (Table 5).

**Table 5:** Spearman’s rank correlation coefficient for physiotherapists’ years of service and their rating of physiotherapy education systems (all correlations are significant with *p* = 0.001)

Characteristics of physiotherapy education systems rated by respondents	Correlation of the rating with years of service	
	Polish physiotherapists	German physiotherapists
**I. Form and content of educational programs**		
Quality of teaching	−0.92	−0.66
Specialties available in physiotherapy courses	− 0.97	− 0.69
Work by the academic stuff	− 0.82	− 0.69
Teaching skills of the academic staff	− 0.80	− 0.93
Curriculum	− 0.93	− 0.64
System of internships	− 0.89	− 0.88
**II. Organisation of education**		
Conditions at the university	−0.86	− 0.87
Opportunity to do part of the course abroad	−0.93	−0.59
Functioning of university library and reading room	−0.94	−0.74
University administration and functioning	−0.94	−0.62
Student facilities at the university	−0.88	−0.61
**III. Effectiveness of education**		
Acquisition of knowledge-related professional qualifications	−0.87	−0.73
Acquisition of skills-related professional qualifications	−0.87	−0.66
Acquisition of professional qualifications in the area of social competences	−0.90	−0.69
Practical training for the profession	−0.86	−0.83
Acquisition of qualifications relative to current needs of labour market	−0.91	−0.84

Perceptions of working conditions and career satisfaction, similar to those related to the process of formal education, are strongly related to respondents’ years of service (all correlations are significant at *p* = 0.001). Negative coefficients of correlation between years of service and perceptions of all the aspects of work show that therapists with more work experience tend to be more critical about their job (Table 6).

**Table 6:** Spearman’s rank correlation coefficient for physiotherapists’ years of service and their rating of work-related issues (all correlations are significant at p = 0.001)

Work-related issues evaluated by respondents	Correlation of the rating with years of service	
	Polish physiotherapists	German physiotherapists
Atmosphere at work	−0.49	−0.92
Satisfying relations with supervisor	−0.66	−0.86
Salary satisfaction	−0.43	−0.80
Job satisfaction	−0.22	−0.47
Career advancement	−0.62	−0.82

To examine this tendency in more detail, regression analysis was carried out taking into account the variables corresponding to participants’ perceptions of the educational process and professional career in relation to their years of service and, additionally, their age.

The effect of the selected factors on self-reported job satisfaction was assessed using linear regression analysis, which showed that the independent predictors of satisfaction with the occupation of physiotherapist included the following:

- Age: each subsequent year of life decreases this rating on average by 0.002 points,
- Years of service: compared to the individuals at the start of their professional career (0 years of service), work experience in the range of 16–20 years increased the rating on average by 0.36 points.

With regard to the assessment of the effect of the selected factors on self-reported career advancement, the linear regression analysis showed that the independent predictors of subjective perceptions of one’s professional career included the following:

- Age: each subsequent year of life decreases this rating on average by 0.001 points,
- Years of service: compared to physiotherapists starting their professional career, those with work experience in the range of 16–20 years increased the rating on average by 1.10 points.

## Discussion

A review of the related literature suggests that no studies thus far have investigated differences in the formal education and professional development of physiotherapists in Poland and Germany. Such research would make it possible to identify positive aspects of the education system in one country and introduce them into the education system in the other country; as a result, it would also be possible to continue to align practice in both countries to the evolving scope of physiotherapy practice and epidemiological needs in an ever-evolving dynamic manner.

The first research problem was related to the assessment of the coherence of the findings about Polish and German physiotherapy education systems based on participants’ perceptions. The acquired findings suggest that the physiotherapy education systems differ in terms of all the evaluated characteristics, and the Polish system received poorer ratings in most aspects. It should be pointed out, however, that the education systems were assessed using a subjective method by individuals living in different socioeconomic environments. Even though these are neighboring countries, Germany and Poland represent different historical and cultural backgrounds, since traditionally they are linked to Western and Eastern Europe, respectively. Generally, the standard of living is much higher in Germany than in Poland, which reflects the different economic statuses of these two countries, with the gross domestic product per capita in 2020 being approximately three times higher in Germany than that in Poland [23]. Therefore, the current findings should be treated as a reflection of the participants’ experiences rather than an objective evaluation of the state of the two educational systems. Both in Poland and in Germany, the curricula defined for university courses in physiotherapy comply with both national requirements and the guidelines of the World Confederation for Physical Therapy [19]. Nevertheless, the present comparative research findings suggest that there is no coherence between physiotherapy education systems in Poland and Germany. Starczyńska et al. conducted a study involving 338 physiotherapists working in Poland and 125 physiotherapy students and assessed their subjective perceptions about the profession in the context of the required competencies. The researchers concluded that the progress in both research skills and professional competencies reflected the advancement achieved by Polish physiotherapy. According to their study participants, the job of the physiotherapist is satisfying; it provides reliability and presents career opportunities. It is, however, necessary to improve the quality of practical education as part of university courses [24]. On the other hand, Gotlib et al. investigated the perception of physiotherapy as a career by students at different types of universities. The study involved 1145 first-year students completing BA courses at eleven Polish universities that provide education in physiotherapy. The investigators also employed an opinion polling method based on a questionnaire. According to the majority of students, the professional status of physiotherapists is lower than the professional status of doctors but higher than the professional status of nurses [25].

The current study also aimed to compare the professional status of physiotherapists in Poland and Germany. Our findings support that German physiotherapists perceive their professional situation as superior to that of their Polish colleagues. Additionally, in this case, no reports were found to be used as a reference in the discussion of the results and in support of the hypothesis. It seems, however, that the findings of a study assessing occupational satisfaction of physiotherapists in West Pomerania, Poland, are interesting in this context. The term was defined as “satisfaction resulting from performance of specific tasks”. The findings showed that the financial factor of occupational satisfaction is directly proportional to the remuneration received at the time when job satisfaction is inhibited [26]. This may be aptly explained by reference to the financial situation in both countries, as it may affect the quality of work performed. Research shows that a high level of occupational satisfaction significantly corresponds to the level of the person’s commitment, diligence, long-term employment and rare absences from work. In the case of health care workers, their attitude toward their occupation significantly affects the quality of services provided as well as patient satisfaction [27]. Likewise, assessment of one’s life situation directly and indirectly affects overall perceptions of one’s professional status. This is illustrated by the mechanism of the close causal relationship between work and material gain. External working conditions, organizational framework, material benefits, occupational prestige, opportunities for professional growth, career advancement, and promotion as well as other elements directly or indirectly affect the progress and effects of one’s occupation [28, 29]. The findings of the present study suggest that the overall job satisfaction and perceptions of one’s general life situation (comprising such factors as being content with one’s job, opportunities for further education, and financial gains from work) are higher in the case of German physiotherapists. We were not able to acquire reliable data about the salaries of physiotherapists in Poland and Germany since the amount of remuneration is confidential under employment contracts. However, a comparison of the average monthly gross salary during the period covered by this study, in Poland amounting to approximately 1080 Euro [30] and in Germany totaling 4100 Euro [31], suggests that the financial situation was far better in the latter country.

The third detailed research problem was related to the professional development of physiotherapists in Poland and Germany and their career opportunities. The survey showed that perceptions of physiotherapy education, as well as perceptions related to working conditions and job satisfaction, are closely linked to the respondents’ years of service. The negative correlation coefficients between years of service and perceptions of all aspects of the occupation provide evidence that therapists with more work experience are more critical about their profession. This may be because as they gain experience, their expectations toward the employer and toward themselves increase. This phenomenon may be associated with a tendency to reflect on one’s life, occurring irrespective of the country of residence. This may also be linked to older physiotherapists’ awareness of the progressive changes taking place in various areas related to their profession, e.g., the inevitable differences in entry-level education between the past and today. This in fact is absolutely understandable and is to be expected. The evolution of curricula, teaching methodology, and the use of evidence-based theories for curricular content corresponds to changes in the scope of physiotherapy practice. These changes are needed and desirable, given that physiotherapy is the leading ‘nonpharmacological and nonsurgical’ health profession with a capacity to provide effective noninvasive treatments to prevent, manage or even reverse the effects of lifestyle-related noncommunicable diseases (NCDs), which in today’s world are the most urgent health problem.

We must also point out that the youngest participants were only 1 year out from graduation, whereas the oldest participants graduated 20 years before the study. This fact definitely explains some differences in perceptions related to entry-level education. The opinions presented by older graduates may be affected by the passing of time and their ability to accurately recall the specific aspects of their professional education. Furthermore, more work experience leads to greater awareness of the actual needs related to one’s profession, which might bring to light the shortcomings of the education acquired at school. Likewise, advances taking place in the course of one’s career may also “infiltrate” one’s opinions about the past. What is most important, however, is the fact that the educational systems and the teaching curricula two decades ago and now are not the same, quite understandably. In 20 years, physiotherapy education has seen dynamic changes in both countries, associated with the development of educational structures and facilities and, more importantly, with increasing supervision of the quality of education and changes in educational curricula, in line with the accreditation standards of World Physiotherapy, of which both countries are members. In fact, we hope that educational curricula in the two countries have changed significantly in the last 10 or even 5 years, taking into account newly acquired evidence and epidemiological needs. Overall, the significant difference in years of service suggests that specific participants have had quite different experiences and opportunities. Some of these factors may also explain differences observed in the perceptions of physiotherapists with more experience.

Our findings support the need to unify the systems of physiotherapy education across countries in the European Union to maintain high standards of care. In addition, comparable education for physiotherapists across the EU would facilitate their movement to practice in other European countries.

### Study limitations

This study presents certain limitations; however, it should be remembered that this is the first research report related to this specific subject matter. We suggest the following ways to expand and enhance further research:

1. Sample representativeness should be improved by applying random selection (to do this it would be necessary to acquire information needed for the sampling frame and then to make sure that the randomly selected individuals provide required information, presumably during personal contact). Notably, in the current study, potential participants decided whether to respond to the invitation to take part in the survey; therefore, their responses may reflect their variable motivations; due to this, the generalizability of the findings is limited.
2. Objective indicators of the assessed variables should be used, e.g., use of other information in addition to the questionnaire.
3. There should be qualitative analysis of the differences in the functioning of therapists (resulting from personal background and social environment).
4. Other countries should be included in the study.

## Conclusions

Our questionnaire-based study, investigating career-related perceptions of Polish and German physiotherapists, showed certain similarities and differences in their perceptions concerning formal education, professional development and career opportunities. Further research is needed to directly compare formal physiotherapy education systems, as well as professional development requirements, mechanisms, and opportunities in Poland and Germany. Comparative analyses of physiotherapists’ perceptions, educational systems, teaching curricula and professional development opportunities in the two countries will provide information to entry-level physiotherapy educators as well as professional organizations to recommend changes in professional education in both countries.

## Data Availability

The datasets generated and/or analyzed during the current study are not publicly available due to privacy considerations but are available from the corresponding author on reasonable request.

## Abbreviation

EU: European Union
